# No insulin degludec dose adjustment required after aerobic exercise for people with type 1 diabetes: the ADREM study

**DOI:** 10.1007/s00125-023-05893-9

**Published:** 2023-03-07

**Authors:** Linda C. A. Drenthen, Mandala Ajie, Evertine J. Abbink, Laura Rodwell, Dick H. J. Thijssen, Cees J. Tack, Bastiaan E. de Galan

**Affiliations:** 1 Department of Internal Medicine, Radboud University Medical Center, Nijmegen, the Netherlands; 2 Department for Health Evidence, Section Biostatistics, Radboud Institute for Health Sciences, Radboud University Medical Center, Nijmegen, the Netherlands; 3 Department of Physiology, Radboud Institute for Health Sciences, Radboud University Medical Center, Nijmegen, the Netherlands; 4 Research Institute for Sport and Exercise Sciences, Liverpool John Moores University, Liverpool, UK; 5 Department of Internal Medicine, Maastricht University Medical Center (MUMC+), Maastricht, the Netherlands

**Keywords:** Dose adjustment, Exercise, Hypoglycaemia, Insulin treatment, Type 1 diabetes mellitus

## Abstract

**Aims/hypothesis:**

It is generally recommended to reduce basal insulin doses after exercise to reduce the risk of post-exercise nocturnal hypoglycaemia. Based on its long *t*_½_, it is unknown whether such adjustments are required or beneficial for insulin degludec.

**Methods:**

The ADREM study (Adjustment of insulin Degludec to Reduce post-Exercise (nocturnal) hypoglycaeMia in people with diabetes) was a randomised controlled, crossover study in which we compared 40% dose reduction (D40), or postponement and 20% dose reduction (D20-P), with no dose adjustment (CON) in adults with type 1 diabetes at elevated risk of hypoglycaemia, who performed a 45 min aerobic exercise test in the afternoon. All participants wore blinded continuous glucose monitors for 6 days, measuring the incidence of (nocturnal) hypoglycaemia and subsequent glucose profiles.

**Results:**

We recruited 18 participants (six women, age 38 ± 13 years, HbA_1c_ 56 ± 8 mmol/mol [7.3 ± 0.8%], mean ± SD). Time below range (i.e. glucose <3.9 mmol/l) the night after the exercise test was generally low and occurrence did not differ between the treatment regimens. During the subsequent whole day, time below range was lower for D40 compared with CON (median [IQR], 0 [0–23] vs 18 [0–55] min, *p*=0.043), without differences in the number of hypoglycaemic events. Time above range (i.e. glucose >10 mmol/l) was greater for D20-P vs CON (mean ± SEM, 584 ± 81 vs 364 ± 66 min, *p*=0.001) and D40 (385 ± 72 min, *p*=0.003).

**Conclusions/interpretation:**

Post-exercise adjustment of degludec does not mitigate the risk of subsequent nocturnal hypoglycaemia in people with type 1 diabetes. Although reducing degludec reduced next-day time below range, this did not translate into fewer hypoglycaemic events, while postponing degludec should be avoided because of increased time above range. Altogether, these data do not support degludec dose adjustment after a single exercise bout.

**Trial registration:**

EudraCT number 2019-004222-22

**Funding:**

The study was funded by an unrestricted grant from Novo Nordisk, Denmark.

**Graphical abstract:**

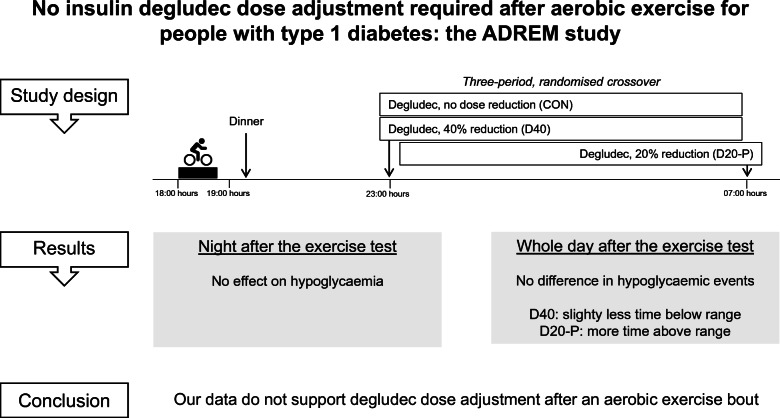

**Supplementary Information:**

The online version of this article (10.1007/s00125-023-05893-9) contains peer-reviewed but unedited supplementary material.

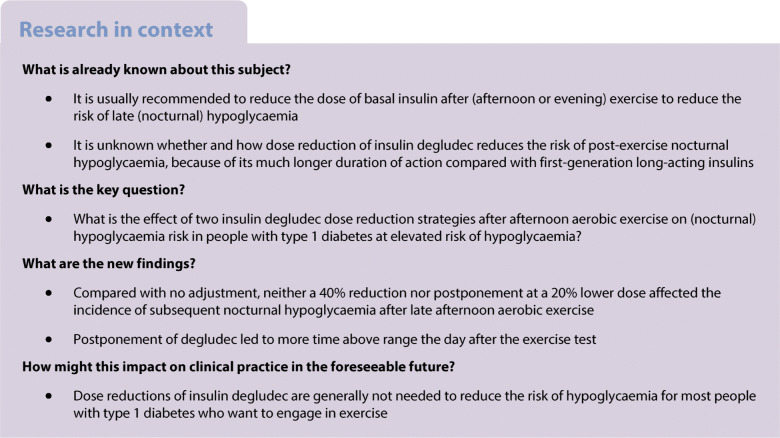



## Introduction

Regular physical exercise is recommended for people with type 1 diabetes mellitus given its beneficial effects on general well-being, cardiometabolic health and insulin requirements [1]. However, aerobic exercise in people with type 1 diabetes increases the risk of hypoglycaemia due to the inability to adjust for falling insulin requirements [2, 3]. This risk is amplified because muscle glycogen storage needs to be replenished, leading to increased insulin sensitivity and glucose disposal. These effects usually peak 7–11 h after exercise, but can last for up to 24 h [4]. As a consequence, there is an increased risk of nocturnal hypoglycaemia, particularly after engaging in sports in the afternoon or evening [4, 5]. This may be even more pronounced in people with reduced awareness of hypoglycaemia. Nocturnal hypoglycaemia is associated with impaired cognitive function and well-being [6] and is the main barrier for people with type 1 diabetes to engage in sports [7]. Dose reduction of meal-related bolus insulin does not prevent late-onset (nocturnal) hypoglycaemia in people using first-generation long-acting insulins [8]. However, reducing these basal insulins after exercise can prevent exercise-induced (nocturnal) hypoglycaemia [9], as can reducing the basal rate of insulin infusion pumps [10]. To mitigate the risk of nocturnal hypoglycaemia, it is therefore typically recommended to reduce the dose of first-generation long-acting insulin at bedtime or the basal rate of insulin infusion in pump users by 20% after afternoon or evening exercise [3].

Insulin degludec is a second-generation long-acting insulin analogue with a much longer *t*_½_ compared with other long-acting insulins, resulting in a more stable glucose-lowering profile and longer duration of action [11, 12]. Use of insulin degludec has been associated with reduced risks of hypoglycaemia, particularly nocturnal events [13]. However, it is suggested that insulin degludec carries the same risk for post-exercise (nocturnal) hypoglycaemia compared with insulin glargine in people with type 1 diabetes [14]. The long *t*_½_ of degludec has important implications for dosing adjustments, since a steady state will be reached no earlier than after 2–3 days [15]. One study found that a 25% dose reduction of insulin degludec did not reduce the risk for hypoglycaemia in people with type 1 diabetes during 5 consecutive days of moderate-intensity activity, but this study was very small (*n*=7) [16]. As such, it is unclear what recommendation for insulin dose reduction after exercise is justified for insulin degludec. Therefore, this study compared the effects of two different degludec dose adjustments with no adjustment on the incidence of nocturnal hypoglycaemia and glucose profiles after aerobic exercise in people with type 1 diabetes at elevated risk of hypoglycaemia.

## Methods

### Study procedures

The ADREM study (Adjustment of insulin Degludec to Reduce post-Exercise (nocturnal) hypoglycaeMia in people with diabetes) was an open-label, randomised controlled, three-way crossover study, conducted in accordance with the principles of the Declaration of Helsinki, the Medical Research Involving Human Subjects Act and applicable International Conference on Harmonization (ICH) Good Clinical Practice guidelines. The study was approved by the local ethics committee and national competent authority. All participants gave their written informed consent before any study-related activity was performed.

### Study participants

Adults aged 18–60 years were eligible for participation when they had been diagnosed with type 1 diabetes for at least 2 years, had been treated with a basal-bolus multi-dose insulin regimen for at least 1 year and were at increased risk of hypoglycaemia. The latter was defined as a history of at least one severe hypoglycaemia event in the past year and/or ≥2 points on the Dutch modified version of the Clarke questionnaire and/or ≥3 points on the Gold score [17–19]. They also had to engage in moderate-intensity exercise for at least 1 h per week and had to have an HbA_1c_ ≤75 mmol/mol (9%). Main exclusion criteria were microvascular complications (except for background retinopathy or a urinary albumin/creatinine of maximum 30 mg/mmol), BMI >30 kg/m^2^, pregnancy, Modification of Diet in Renal Disease (MDRD) GFR <60 ml/min per 1.73 m^2^, any contraindication for exercise testing according to the American Heart Association (AHA) guidelines [20] and the use of β-blockers or drugs affecting glucose metabolism other than insulin. Participants were recruited from the outpatient clinic of the Radboud University Medical Center and Rijnstate Hospital and websites of patient associations.

### Screening visit

**Fig. 1:**
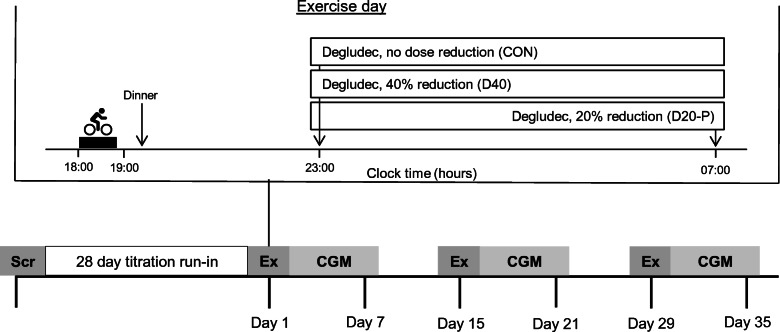
Overview of the study. Ex, exercise day; Scr, screening visit

A schematic overview of the study design is shown in Fig. 1. All participants performed an incremental cardiopulmonary exercise test (CPET) (Lode Excalibur; Lode, Groningen, the Netherlands) on a bicycle ergometer to determine their maximum cardiovascular fitness level (defined as $\dot{V}{\mathrm{O}}_{2\max }$) and maximum heart rate [21]. During CPET, the participant was asked to cycle at a continuous rate of 60 to 80 rotations per minute and the work rate was increased every minute by 15 or 20 W until exhaustion, or an indication to stop according to the American Thoracic Society/American College of Chest Physicians (ATS/ACCP) statement [22]. Directly before and after cycling, venous blood was collected to measure glucose and lactate concentrations using Biosen C-Line (EKF Diagnostics, Cardiff, UK). After screening, all participants were instructed to inject insulin degludec at 23:00 hours during the trial, and participants not on degludec were transferred to it. A 28 day titration run-in was used to reach stable glycaemic control, defined as a self-measured fasting mean glucose concentration below 7 mmol/l.

### Exercise days

Each participant engaged in 3 exercise days and was randomly assigned by the investigator to an order of the three post-exercise degludec treatment regimens, i.e. no adjustment of insulin degludec (CON), a 40% dose reduction of degludec (D40) and 8 h postponement with a 20% dose reduction of degludec (D20-P). For D40, the usual recommended long-acting insulin dose reduction of 20% was doubled, because degludec has a *t*_½_ of about twice that of insulin glargine. This means that, in theory, plasma insulin levels will gradually fall and be 20% lower at the time of the next injection, and ~7% lower the next morning. By postponing the injection by 8 h (i.e. a third of the *t*_½_), we expected insulin levels to fall ~16% overnight. The six potential treatment sequences were evenly distributed among the trial population. The exercise days were each separated by a period of 14 days (±3 days), except that 5 exercise days had to be postponed for up to 14 days because of COVID-19 restrictions. Every exercise day was followed by 6 days of blinded continuous glucose monitoring (CGM) (Dexcom G6; Dexcom, San Diego, CA, USA). Participants were also allowed to simultaneously use their own glucose sensor. Sleep times were recorded using an activity tracker (activPAL3 micro; PAL Technologies, Glasgow, UK) [23]. Participants were requested to refrain from strenuous exercise of all types during the 2 days before and 7 days after the exercise tests. On the exercise days, participants consumed their lunch at home with a 50% dose reduction of their short-acting insulin to prevent hypoglycaemia before and during the exercise test. Between 15:30 and 16:30 hours, participants arrived at the research facility, where CGM was started. Blood was sampled for determination of glucose, lactate, insulin, catecholamines and cortisol at arrival, 5 min before and after the exercise test, and before discharge. Depending on the participant’s glucose concentration and its trend before the exercise test, the participants consumed a carbohydrate-rich snack aiming for a blood glucose concentration of 7–14 mmol/l. At 18:00 hours, participants performed a 45 min exercise test on a bicycle ergometer at 70% of their heart rate reserve using the Karvonen formula, based on the maximum heart rate determined by CPET and the resting heart rate measured during the screening visit [24]. During the exercise test, the participant’s glucose concentration was monitored by measuring their interstitial glucose level using their own glucose sensor and additional carbohydrates were given when necessary. After the exercise test and before discharge, participants consumed a standardised meal (consisting of 45–50% carbohydrates, 30–40% protein and 20–30% fat) with a 25% dose reduction of their short-acting insulin. Participants were instructed not to eat from discharge until getting up the next day, except in case of hypoglycaemia. They were also instructed not to inject any short-acting insulin from discharge until getting up the next day, except in case of profound hyperglycaemia. At 23:00 hours on the exercise day (CON and D40) or 07:00 hours the next day (D20-P), the participants administered insulin degludec. The day after the exercise tests, participants measured their fasting ketones by point-of-care testing before 07:30 hours. They also registered their injected insulin dose for 6 days after the exercise day.

### Study outcomes

The primary outcome was the time below range (i.e. glucose <3.9 mmol/l) in the night (00:00–05:59 hours) following the exercise test. Secondary outcomes included times above range (i.e. glucose >10.0 mmol/l) and in range (i.e. glucose ≥3.9 mmol/l and ≤10.0 mmol/l), mean glucose concentration, number of hypoglycaemic and severe hypoglycaemic (requiring external assistance for recovery) events and total daily dose of short-acting insulin. All outcome variables were calculated during the first and second days (00:00–23:59 hours) after the exercise test as well as for the total 6 days following the exercise test. A hypoglycaemic event was defined as a glucose concentration <3.9 mmol/l for at least 15 consecutive minutes and a new event was calculated if the glucose concentration had been risen above this level for at least 15 min [25]. All CGM outcomes were calculated using R version 4.1.2 (R Foundation for Statistical Computing, Vienna, Austria).

### Measurements

Plasma insulin was measured using an in-house radioimmunoassay, using an in-house-generated guinea pig anti-human insulin antibody and ^125^I-labelled human insulin tracer. ^125^I-labelled human insulin tracer was generated using ^125^I (PerkinElmer Nederland) and human insulin (Novo Biolabs cat. no. 471). In this assay, bound–free separation is performed by second antibody/polyethylene glycol precipitation of antibody-bound insulin. The assay is calibrated on World Health Organization international standard 83/500. The cross-reactivity in this method is approximately 60% for insulin aspart and 50% for insulin lispro. The cross-reactivity for insulin degludec is not well known. Catecholamines were analysed by an LC-MS/MS method developed and validated in-house after derivatisation with propionic anhydride and subsequent solid-phase extraction [26]. Plasma cortisol was determined using a routine analysis method with an electrochemiluminescent immunoassay on a Cobas E801 random access analyser (Roche Diagnostics, Mannheim, Germany) [27].

### Statistical analyses

The sample size estimation was based on the two treatments for which the smallest difference was expected (D40 and CON). Given this was a crossover trial, it was expected that the other comparisons (with D20-P) would have sufficient power. No ɑ corrections were made to account for the multiple comparisons. Furthermore, it was assumed that the within-person correlation for the response measures on the different treatments was 0.65. We aimed at finding a significant decrease in time below range with at least 20% increase in time in range during the night after the exercise test and 50% increase in next-morning fasting glucose concentration [9]. We calculated that 13 participants would be required to detect a difference at a significance level of 0.05 and a power of 80%. To account for the relatively small number of participants involved, a total of 18 participants were enrolled. Data were analysed using IBM SPSS version 25 and Stata version 16. We performed an as-treated analysis. We used random effects models to account for the three measurements for each participant, with period and treatment as independent variables. Given the low incidence of hypoglycaemia the night after the exercise test, the primary outcome was transformed to a binary outcome to represent no or any time below range, and analysed using a logistic random effects model. Differences in continuous variables between the three treatment arms were analysed using a multilevel mixed-effects linear regression model performing restricted maximum-likelihood estimation. Differences in count data between the study arms were analysed using a negative binomial random effects model. Data that were not normally distributed were log transformed or analysed using the related samples Friedman’s two-way analysis. No adjustments were made to account for multiple testing of the secondary endpoints. Every day started at midnight and the night period was defined as 00:00 hours to 05:59 hours. We performed a sensitivity analysis where we repeated all analyses for the CGM data based on the sleep times of the participants instead of the predefined day and night periods. All data are expressed as mean ± SEM or median [IQR], unless otherwise specified. A *p* value <0.05 was considered statistically significant.

## Results

A total of 19 participants were screened, 18 of whom were included. One participant was withdrawn after screening because of personal reasons unrelated to the study. All 18 included participants completed the study. Their baseline characteristics are shown in Table 1. Nine participants were already on insulin degludec; the other participants were transferred to it with a mean ± SD dose of 87 ± 10% of their pre-study long-acting insulin dose (insulin glargine and detemir). There were no differences in the proportion of glucose readings by the glucose sensor between the treatment regimens during the total 6 day periods (mean ± SD, CON 99 ± 2%, D40 95 ± 16%, D20-P 98 ± 3%). One participant was on real-time CGM and 17 used flash-glucose monitoring, three of whom had the alarm function for low and high glucose concentrations turned on. All participants achieved maximal exhaustion during CPET (electronic supplementary material [ESM] Table 1). No serious adverse events occurred during the study. One participant had mild cellulitis on her foot and one participant was infected by COVID-19 during the study period, but neither were judged to be related to the study, nor to have impact on the study results.

**Table 1:** Baseline characteristics

Characteristic	*n*=18
Age, years	38 ± 13
Male sex	12 (67)
BMI, kg/m^2^	25.0 ± 2.7
Duration of diabetes, years	12 ± 11
HbA_1c_, mmol/mol	56 ± 8
HbA_1c_, %	7.3 ± 0.8
Total insulin dose, U/day	49 ± 26
Short-acting insulin	
Insulin aspart	15 (83)
Fast-acting insulin aspart	2 (11)
Insulin lispro	1 (6)
Score on modified Clarke questionnaire	2 [2–2]
Gold score	2 [2–3]
IAH	5 (28)
Serum creatinine, μmol/l	72 ± 16
$\dot{V}{\mathrm{O}}_{2\max }$, ml min^−1^ kg^−1^	40.2 ± 9.6

Data are presented as number (%), mean ± SD or median [IQR]

IAH, impaired awareness of hypoglycaemia, i.e. ≥3 points on Clarke questionnaire and/or ≥4 points on Gold score

### Exercise tests

The three 45 min exercise tests were performed consistently across all treatment groups regarding heart rate (CON 144 ± 3, D40 145 ± 3, D20-P 144 ± 3 beats per minute). All participants cycled at 70% of their heart rate reserve, except for one participant, who cycled all three tests at 57% heart rate reserve because of mild ST-segment depression during CPET. The increase in blood lactate (Δ lactate CON 0.76 ± 0.15, D40 0.97 ± 0.35, D20-P 0.69 ± 0.14 mmol/l) and decrease in blood glucose levels (Δ glucose CON −3.88 ± 0.95, D40 −4.91 ± 0.50, D20-P −3.69 ± 0.58 mmol/l) during cycling did not differ for the three exercise tests (ESM Fig. 1). Six participants in CON, three in D40 and six in D20-P ingested additional carbohydrates shortly before or during the test to prevent hypoglycaemia. The counter-regulatory hormone responses to exercise did not differ between treatment regimens, except for the cortisol response which was slightly higher during D40 compared with CON (97 ± 37 vs 53 ± 39 nmol/l, *p*=0.03) (ESM Fig. 1).

### Study outcomes

#### Time below range and hypoglycaemic events

Time below range in the night after the exercise test was generally low and occurrence did not differ significantly between the treatment regimens (three participants in CON, one in D40, three in D20-P). The day after the exercise test, time below range was greater for CON compared with D40 (18 [0–55] vs 0 [0–23] min, *p*=0.043) (ESM Fig. 2), but the number of hypoglycaemic events was similar (ESM Table 2). During the second whole day, D20-P was associated with more time below range (28 [4–46] vs 0 [0–41] min, *p*=0.019) and more hypoglycaemic events (20 vs 9, *p*=0.027) compared with D40, but neither differed significantly from CON (5 [0–44] min; 13 events). No differences in time below range and hypoglycaemic events were found between the treatment regimens during the total 6 days after the exercise test. No severe hypoglycaemic events occurred during the entire study period.

#### Mean glucose concentration

**Fig. 2:**
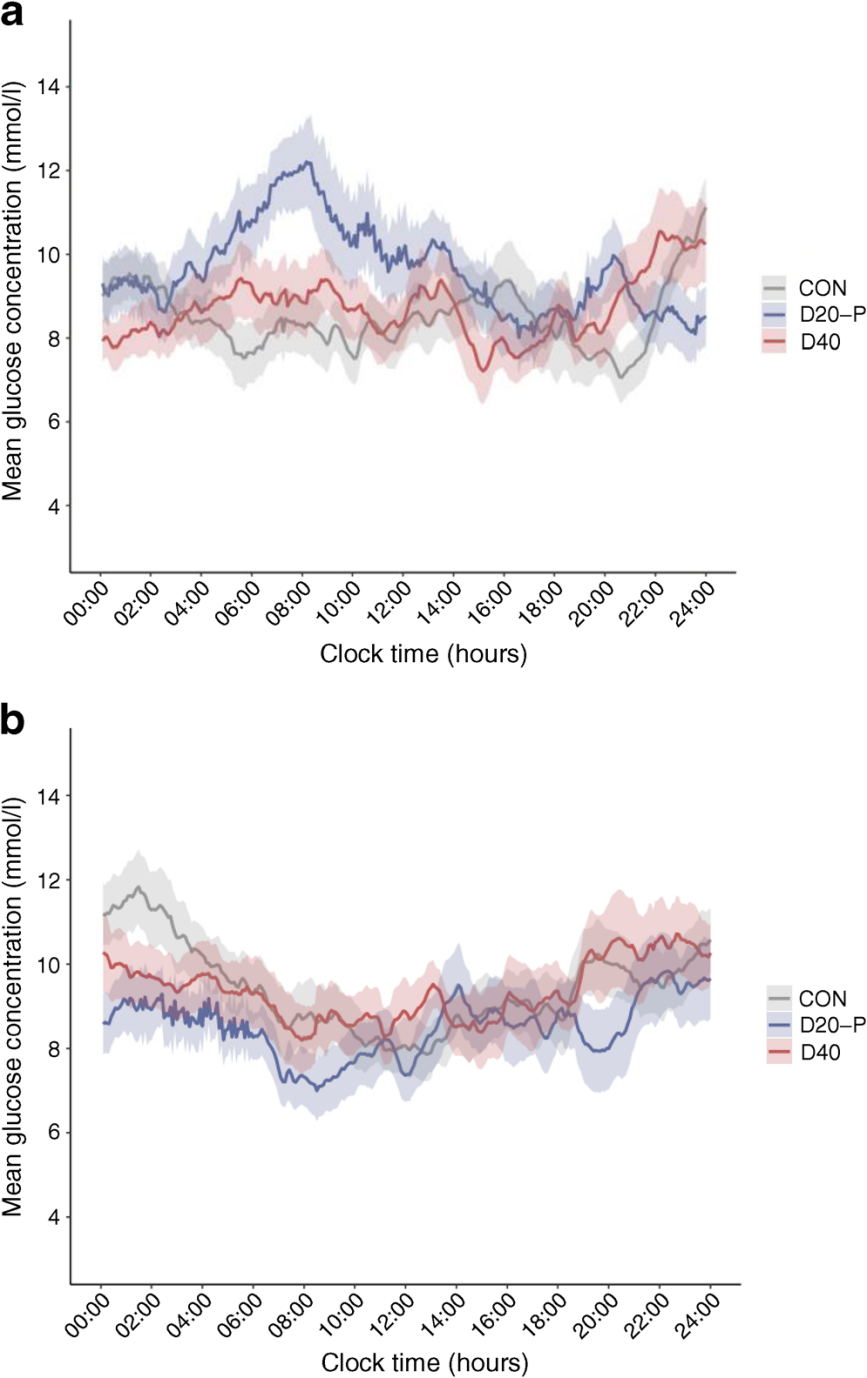
Time course of the mean glucose concentration over the first (**a**) and second day (**b**) after the exercise test, according to insulin degludec dosing regimen. Grey, CON; red, D40; blue, D20-P. Values are given as mean ± SEM

No differences in mean glucose concentration were found between the treatment regimens the night after the exercise test (Fig. 2). The day after the exercise test, D20-P was associated with a higher mean glucose concentration (9.6 ± 0.5 mmol/l) compared with CON (8.5 ± 0.4 mmol/l, *p*=0.015) and D40 (8.7 ± 0.5 mmol/l, *p*=0.035) (ESM Table 2). The second day after the exercise test, D20-P was associated with a lower mean glucose concentration compared with CON (8.6 ± 0.5 vs 9.8 ± 0.5 mmol/l, *p*=0.014), but neither differed significantly from D40 (9.5 ± 0.5 mmol/l). No differences in mean glucose concentration were found between the treatment regimens during the total 6 days after the exercise test.

#### Time above range

**Fig. 3:**
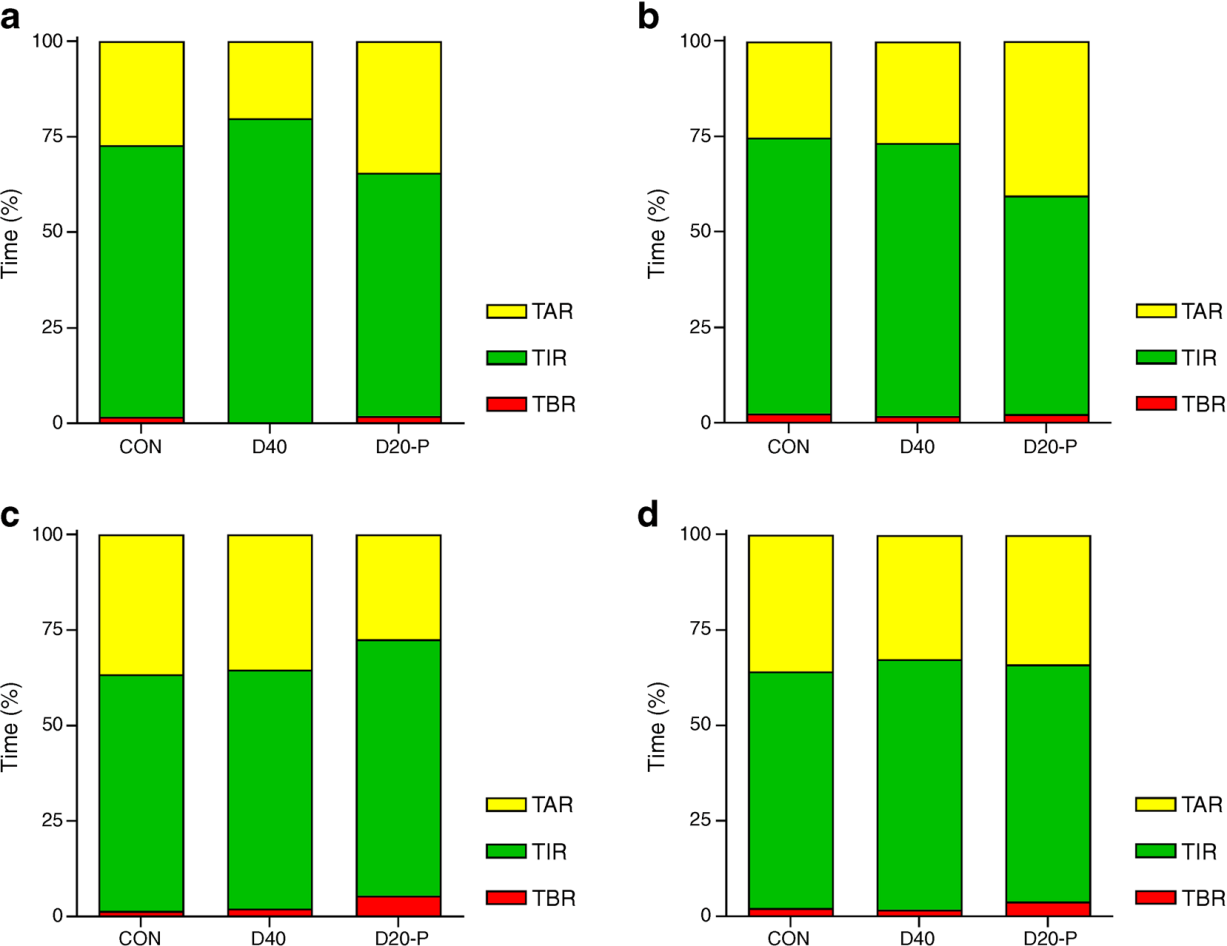
Percentage of time spent above range, in range and below range the first night (**a**), first day (**b**), second day (**c**) and total 6 days (**d**) after the exercise test, according to insulin degludec dosing regimen. TAR, time above range (yellow); TIR, time in range (green); TBR, time below range (red). Data are given as mean values

No differences in time above range were found between the treatment regimens the night after the exercise test. The day after the exercise test, D20-P led to significantly more time above range (584 ± 81 min) compared with CON (364 ± 66 min, *p*=0.001) and D40 (385 ± 72 min, *p*=0.003) (Fig. 3). No differences in time above range were found between the treatment regimens during the second day after the exercise test, nor for the total 6 day period.

#### Time in range

The night following the exercise test, D20-P was associated with less time in range compared with D40 (229 ± 30 vs 287 ± 26 min, *p*=0.027), but neither differed significantly from CON (256 ± 26 min). The day after the exercise test, D20-P led to significantly less time in range (824 ± 74 min) compared with CON (1041 ± 62 min, *p*=0.001) and D40 (1029 ± 70 min, *p*=0.002). No differences in time in range were found between the treatment regimens during the second day after the exercise day, nor for the total 6 day period.

#### Sensitivity analyses

Repeating the analyses according to the actual sleep times of the participants did not materially change the results, except that the night after the exercise test, the mean glucose concentration was higher for D20-P compared with CON (9.8 ± 0.8 vs 8.5 ± 0.5 mmol/l, *p*=0.044), but neither differed significantly from D40 (8.9 ± 0.7 mmol/l) (ESM Table 3). Furthermore, during this night, time in range was lower for D20-P when compared with both D40 (255 ± 44 vs 366 ± 46 min, *p*=0.014) and CON (376 ± 35 min, *p*=0.008). The day after the exercise test, the mean glucose concentration was higher for D20-P only when compared with CON (9.6 ± 0.5 vs 8.4 ± 0.4 mmol/l, *p*=0.012), whereas neither differed significantly from D40 (8.8 ± 0.5 mmol/l). The second day after the exercise test, time below range was higher for D20-P when compared with both D40 (28 [4–65] vs 0 [0–41] min, *p*=0.016) and CON (5 [0–44] min, *p*=0.038).

#### Fasting ketones

Fasting ketones in the morning after the exercise tests were generally low (all ≤0.8 mmol/l), but were significantly higher for D20-P than D40 (0.27 ± 0.04 vs 0.16 ± 0.03 mmol/l, *p*=0.022), neither of which differed from CON (0.21 ± 0.05 mmol/l).

#### Short-acting insulin and carbohydrate intake

The evening after the exercise test, three people (17%) in CON, one (6%) in D40 and one (6%) in D20-P injected additional short-acting insulin because of profound hyperglycaemia. During this evening, nine people (50%) in CON, five (28%) in D40 and two (11%) in D20-P ingested additional carbohydrates to prevent hypoglycaemia. However, of these participants, only one within each treatment arm ingested carbohydrates after 23:00 hours without experiencing a nocturnal hypoglycaemic event. No differences were found in the total daily dose of short-acting insulin used between the treatment regimens on the first and second days after the exercise test (ESM Fig. 3).

## Discussion

The main finding of this study is that adjustment of insulin degludec dosing after aerobic exercise performed in the afternoon had no effect on the incidence of subsequent nocturnal hypoglycaemia in people with type 1 diabetes. While next-day time below range was slightly reduced in the 40% dose reduction group, this did not translate to fewer hypoglycaemic events. Postponement of degludec to the next morning at a 20% lower dose led to more time above range and less time in range during that day, as well as slightly more time below range on the subsequent second day. Altogether, these results do not support dose adjustments of degludec in people with type 1 diabetes after afternoon aerobic exercise.

Two recent studies have reported a relatively low incidence of nocturnal hypoglycaemia after aerobic exercise in people with type 1 diabetes using insulin degludec [16, 28]. We extend those findings by showing that this is also the case for people at elevated risk for hypoglycaemia. We believe that meticulous adherence to the protocol of short-acting insulin dose reductions at the subsequent meal after exercise was critical for this result. Although this is not sufficient for people using first-generation long-acting insulins [8, 29], reducing the meal-related dose of short-acting insulin after evening exercise may reduce the risk of nocturnal hypoglycaemia in people with type 1 diabetes on insulin degludec [28]. This could be due to the more durable and stable pharmacodynamic profile of insulin degludec when administered at fixed timepoints as compared with first-generation long-acting insulins [13].

Heise et al reported insulin glargine and insulin degludec to have a similar (nocturnal) hypoglycaemic risk profile after performing aerobic exercise of moderate intensity in people with type 1 diabetes, without insulin dose reductions [14]. However, patients at high risk for hypoglycaemia were excluded from participation and participants injected their long-acting insulin in the morning. Since insulin glargine has the strongest glucose-lowering effect during the first 12 h after injection [11], a higher number of nocturnal hypoglycaemic events is plausible for people injecting this type of insulin before bedtime, as is still common practice. In addition, the blood glucose concentration was measured at a few predefined timepoints instead of using CGM, so the occurrence of hypoglycaemia may have been underestimated.

For people with type 1 diabetes, the risk of hypoglycaemia is increased for at least 24 h after aerobic exercise, in particular, when performed in the afternoon [5]. Indeed, half of the participants in CON had at least one episode of time below range the day after the exercise test. Although the number of hypoglycaemic events was not reduced, our data suggest that a 40% dose reduction is needed to reduce next-day time below range, without a concomitant increase in the risk of hyperglycaemia. However, these data seem to contrast with general recommendations to reduce the basal insulin component of insulin regimens by 20% after exercise to achieve this result, but may be specific for insulin degludec [3]. Indeed, a 20% dose reduction in the postponement study arm did not lower the risk of subsequent hypoglycaemia. This is supported by previous research showing that a 25% dose reduction of insulin degludec on 5 consecutive days did not protect against hypoglycaemia during the first 48 h after exercise in people with type 1 diabetes [16].

We chose postponement of insulin degludec as one of the dosing adjustment regimens because of previous data showing that alternating degludec dosing at flexible intervals of 8 to 40 h provided about similar glycaemic control to dosing every 24 h [30]. However, in contrast to that study, we found that our participants spent more time above range and less time in range the day after the exercise test when randomised to the postponement study arm, as compared with the other two study arms. The largest difference in mean glucose concentration was seen in the early morning (Fig. 2a), where the additional dose reduction had no effect yet. One explanation for this apparent discrepancy may be the relatively low insulin dose used in our study, since the duration of action of insulin is dose-dependent, with a longer duration of action with larger doses [31, 32]. Indeed, on average, our participants used insulin degludec at a daily dose of 23 units, approximately 10 units less than in the study by Mathieu et al [30]. Nevertheless, our data argue against postponing insulin degludec to reduce post-exercise hypoglycaemia, particularly when insulin doses are low.

Strengths of our study are the randomised crossover study design, the robust and highly reproducible exercise protocol, the use of CGM and the daily life setting, all of which are relevant in the context of the potential need for dose adjustments.

Our study also has limitations. First, it may be questioned to what extent our results can be generalised to people performing morning exercise or injecting degludec in the morning. However, morning exercise leads to a lower risk of late-onset hypoglycaemia compared with afternoon exercise in people with type 1 diabetes on insulin pump therapy [5]. It is similarly unlikely that morning degludec administration is associated with greater risk for nocturnal hypoglycaemia than evening administration. Therefore, we expect that aerobic exercise in the morning can be safely performed by people with type 1 diabetes on insulin degludec without adjusting the dose and irrespective of injection time. Second, more people in CON ingested carbohydrates in the evening after exercise compared with the other two treatment arms. It could be that participants felt unease in breaking their routine of ingesting carbohydrates after exercise in the control arm, even though we advised against it. Although this may have affected the risk of hypoglycaemia in the first couple of hours, additional intake of carbohydrates after exercise has been found to be insufficient for preventing late-night hypoglycaemia in people using first-generation long-acting insulins [29]. In our study, only three people ingested additional carbohydrates after 23:00 hours, which was evenly distributed across the treatment regimens. Besides, slightly more people in CON injected short-acting insulin that evening because of hyperglycaemia, making it further unlikely that late-evening eating played an important role. Third, three people used the automatic hypoglycaemia alarm function of their glucose sensor; because all three had overt impaired awareness of hypoglycaemia, we deemed it unsafe and unethical for them to turn the alarm function off for the sake of the study. However, alarm function settings were similar for all study periods and all three participants spent time below range during every study period with alarms going off to a similar extent. Finally, using a crossover study design has the potential risk of a carry-over or period effect. To minimise these risks, we used block-randomisation and a wash-out period of 2 weeks between the exercise days. In addition, for our statistical analyses we corrected for a period effect, although we would not expect that to be present.

In conclusion, adjustment of insulin degludec dosing after aerobic exercise in the late afternoon has no effect on subsequent nocturnal hypoglycaemia in people with type 1 diabetes. In fact, postponing the administration of degludec leads to more time above range, which underscores the importance of adhering to insulin degludec dosing around exercise, especially when insulin doses are low. Adjustments in meal-related short-acting insulin both before and after exercise may be advisable for people using degludec, but our data do not provide support for standard insulin degludec dose adjustment after exercise in people with type 1 diabetes. These data add evidence for the ease of use of insulin degludec for most people with type 1 diabetes who want to engage in aerobic exercise.

## Supplementary information

ESM (PDF 450 kb)

## Data Availability

The datasets generated during and/or analysed in the current study are available from the corresponding author upon reasonable request.

## Abbreviations

ADREM: Adjustment of insulin Degludec to Reduce post-Exercise (nocturnal) hypoglycaeMia in people with diabetes
CGM: Continuous glucose monitoring
CON: No adjustment of insulin degludec
CPET: Cardiopulmonary exercise test
D40: 40% dose reduction of degludec
D20-P: 8 h postponement with a 20% dose reduction of degludec

## References

[1] Chimen M, Kennedy A, Nirantharakumar K, Pang TT, Andrews R, Narendran P (2012). What are the health benefits of physical activity in type 1 diabetes mellitus? A literature review. Diabetologia.

[2] Metcalf KM, Singhvi A, Tsalikian E (2014). Effects of moderate-to-vigorous intensity physical activity on overnight and next-day hypoglycemia in active adolescents with type 1 diabetes. Diabetes Care.

[3] Riddell MC, Gallen IW, Smart CE (2017). Exercise management in type 1 diabetes: a consensus statement. Lancet Diabetes Endocrinol.

[4] McMahon SK, Ferreira LD, Ratnam N (2007). Glucose requirements to maintain euglycemia after moderate-intensity afternoon exercise in adolescents with type 1 diabetes are increased in a biphasic manner. J Clin Endocrinol Metab.

[5] Gomez AM, Gomez C, Aschner P (2015). Effects of performing morning versus afternoon exercise on glycemic control and hypoglycemia frequency in type 1 diabetes patients on sensor-augmented insulin pump therapy. J Diabetes Sci Technol.

[6] Graveling AJ, Frier BM (2017). The risks of nocturnal hypoglycaemia in insulin-treated diabetes. Diabetes Res Clin Pract.

[7] Brazeau AS, Rabasa-Lhoret R, Strychar I, Mircescu H (2008). Barriers to physical activity among patients with type 1 diabetes. Diabetes Care.

[8] Campbell MD, Walker M, Trenell MI (2013). Large pre- and postexercise rapid-acting insulin reductions preserve glycemia and prevent early- but not late-onset hypoglycemia in patients with type 1 diabetes. Diabetes Care.

[9] Campbell MD, Walker M, Bracken RM (2015). Insulin therapy and dietary adjustments to normalize glycemia and prevent nocturnal hypoglycemia after evening exercise in type 1 diabetes: a randomized controlled trial. BMJ Open Diabetes Res Care.

[10] Taplin CE, Cobry E, Messer L, McFann K, Chase HP, Fiallo-Scharer R (2010). Preventing post-exercise nocturnal hypoglycemia in children with type 1 diabetes. J Pediatr.

[11] Heise T, Hövelmann U, Nosek L, Hermanski L, Bøttcher SG, Haahr H (2015). Comparison of the pharmacokinetic and pharmacodynamic profiles of insulin degludec and insulin glargine. Expert Opin Drug Metab Toxicol.

[12] Heise T, Hermanski L, Nosek L, Feldman A, Rasmussen S, Haahr H (2012). Insulin degludec: four times lower pharmacodynamic variability than insulin glargine under steady-state conditions in type 1 diabetes. Diabetes Obes Metab.

[13] Lane W, Bailey TS, Gerety G (2017). Effect of insulin degludec vs insulin glargine U100 on hypoglycemia in patients with type 1 diabetes: the SWITCH 1 randomized clinical trial. JAMA.

[14] Heise T, Bain SC, Bracken RM (2016). Similar risk of exercise-related hypoglycaemia for insulin degludec to that for insulin glargine in patients with type 1 diabetes: a randomized cross-over trial. Diabetes Obes Metab.

[15] Heise T, Korsatko S, Nosek L (2016). Steady state is reached within 2-3 days of once-daily administration of degludec, a basal insulin with an ultralong duration of action. J Diabetes.

[16] Moser O, Eckstein ML, Mueller A (2019). Reduction in insulin degludec dosing for multiple exercise sessions improves time spent in euglycaemia in people with type 1 diabetes: A randomized crossover trial. Diabetes Obes Metab.

[17] Clarke WL, Cox DJ, Gonder-Frederick LA, Julian D, Schlundt D, Polonsky W (1995). Reduced awareness of hypoglycemia in adults with IDDM. A prospective study of hypoglycemic frequency and associated symptoms. Diabetes Care.

[18] Gold AE, MacLeod KM, Frier BM (1994). Frequency of severe hypoglycemia in patients with type I diabetes with impaired awareness of hypoglycemia. Diabetes Care.

[19] Geddes J, Wright RJ, Zammitt NN, Deary IJ, Frier BM (2007). An evaluation of methods of assessing impaired awareness of hypoglycemia in type 1 diabetes. Diabetes Care.

[20] Gibbons RJ, Balady GJ, Beasley JW (1997). ACC/AHA Guidelines for Exercise Testing. A report of the American College of Cardiology/American Heart Association Task Force on Practice Guidelines (Committee on Exercise Testing). J Am Coll Cardiol.

[21] Janssen L, Frambach S, Allard NAE (2019). Skeletal muscle toxicity associated with tyrosine kinase inhibitor therapy in patients with chronic myeloid leukemia. Leukemia.

[22] Ross RM (2003). ATS/ACCP statement on cardiopulmonary exercise testing. Am J Respir Crit Care Med.

[23] Ryan CG, Grant PM, Tigbe WW, Granat MH (2006). The validity and reliability of a novel activity monitor as a measure of walking. Br J Sports Med.

[24] Karvonen MJ, Kentala E, Mustala O (1957). The effects of training on heart rate; a longitudinal study. Ann Med Exp Biol Fenn.

[25] Battelino T, Danne T, Bergenstal RM (2019). Clinical targets for continuous glucose monitoring data interpretation: recommendations from the international consensus on time in range. Diabetes Care.

[26] van Faassen M, Bischoff R, Eijkelenkamp K, de Jong WHA, van der Ley CP, Kema IP (2020). In matrix derivatization combined with LC-MS/MS results in ultrasensitive quantification of plasma free metanephrines and catecholamines. Anal Chem.

[27] van Meijel LA, Tack CJ, de Galan BE (2021). Effect of short-term use of dapagliflozin on impaired awareness of hypoglycaemia in people with type 1 diabetes. Diabetes Obes Metab.

[28] McCarthy O, Deere R, Churm R (2021). Extent and prevalence of post-exercise and nocturnal hypoglycemia following peri-exercise bolus insulin adjustments in individuals with type 1 diabetes. Nutr Metab Cardiovasc Dis.

[29] Campbell MD, Walker M, Trenell MI (2014). A low-glycemic index meal and bedtime snack prevents postprandial hyperglycemia and associated rises in inflammatory markers, providing protection from early but not late nocturnal hypoglycemia following evening exercise in type 1 diabetes. Diabetes Care.

[30] Mathieu C, Hollander P, Miranda-Palma B (2013). Efficacy and safety of insulin degludec in a flexible dosing regimen vs insulin glargine in patients with type 1 diabetes (BEGIN: Flex T1): a 26-week randomized, treat-to-target trial with a 26-week extension. J Clin Endocrinol Metab.

[31] Walsh J, Roberts R, Heinemann L (2014). Confusion regarding duration of insulin action: a potential source for major insulin dose errors by bolus calculators. J Diabetes Sci Technol.

[32] Plank J, Bodenlenz M, Sinner F (2005). A double-blind, randomized, dose-response study investigating the pharmacodynamic and pharmacokinetic properties of the long-acting insulin analog detemir. Diabetes Care.

