# Supplementary material for: No insulin degludec dose adjustment required after aerobic exercise for people with type 1 diabetes: the ADREM study
Source: Diabetologia. 2023 Mar 7;66(6):1035–44. doi: 10.1007/s00125-023-05893-9 (PMC9988601; doi:10.1007/s00125-023-05893-9)
Supplement: Supplementary file 1 — (PDF 450 kb) [file 125_2023_5893_MOESM1_ESM.pdf]

Electronic supplementary material (ESM)

ESM Table 1. End criteria for maximal exercise testing.

	**n = 18**
$Lactate_{post}$ (mmol/l)	11.26 ± 3.79
RER (ratio)	1.15 ± 0.05
$VO_{2\text{-}diff}$ (ml/min)	82 ± 88
$HR_{max\text{-}pred}$ (beats/min)	182 ± 9
$HR_{max\text{-}test}$ (beats/min)	178 ± 16
$HR_{max\text{-}diff}$ (beats/min)	4 ± 10

$Lactate_{post}$: venous lactate concentration after the test, RER: respiratory exchange ratio, $VO_{2\text{-}diff}$: difference in mean oxygen consumption between the last and before last half-minute of the test, $HR_{max\text{-}pred}$: predicted maximum heart according to Tanaka, $HR_{max\text{-}test}$: heart rate at the end of the test, $HR_{max\text{-}diff}$: difference between the predicted maximum heart and heart rate at the end of the test. Data are presented as mean±SD

ESM Table 2. Glycaemic parameters after the exercise test based on the predefined day and night period.

	**CON**	**D40**	**D20-P**
**1st night after the exercise test**			
Mean glucose concentration	8.7 ± 0.6	8.5 ± 0.6	9.6 ± 0.7
Time below range	0 [0-0]	0 [0-0]	0 [0-0]
Time in range	256 ± 26	287 ± 26	229 ± 30†
Time above range	98 ± 27	72 ± 27	124 ± 31
Hypoglycaemic events	3	1	1
**1st whole day after the exercise test**			
Mean glucose concentration	8.5 ± 0.4	8.7 ± 0.5	9.6 ± 0.5*†
Time below range	18 [0-55]	0 [0-23]*	0 [0-16]
Time in range	1041 ± 62	1029 ± 70	824 ± 74*†
Time above range	364 ± 66	385 ± 72	584 ± 81*†
Hypoglycaemic events	11	8	10
**2nd whole day after the exercise test**			
Mean glucose concentration	9.8 ± 0.5	9.5 ± 0.5	8.6 ± 0.5*
Time below range	5 [0-44]	0 [0-41]	28 [4-46]†
Time in range	892 ± 78	902 ± 92	966 ± 64
Time above range	528 ± 82	509 ± 94	394 ± 70
Hypoglycaemic events	13	9	20†
**Total 6 days after the exercise test**			
Mean glucose concentration	9.5 ± 0.4	9.4 ± 0.4	9.2 ± 0.4
Time below range	38 [15-164]	45 [19-230]	95 [40-370]
Time in range	5345 ± 371	5433 ± 435	5338 ± 321
Time above range	3110 ± 406	2729 ± 417	2938 ± 355
Hypoglycaemic events	61	58	91

Mean glucose concentration in mmol/l, times ranges in minutes, hypoglycaemic events as total count. CON, no dose adjustment; D40, 40% dose reduction; D20-P, postponement and 20% dose reduction. *$p<0.05$ vs CON; †$p<0.05$ vs D40. Data are presented as mean±SEM, median[IQR] or total count (n).

ESM Table 3. Glycaemic parameters after the exercise test based on the actual sleep times.

	**CON**	**D40**	**D20-P**
**1st night after the exercise test**			
Mean glucose concentration	8.5 ± 0.5	8.9 ± 0.7	9.8 ± 0.8*
Time below range	0 [0-4]	0 [0-0]	0 [0-0]
Time in range	376 ± 35	366 ± 46	255 ± 44*†
Time above range	115 ± 34	142 ± 46	199 ± 45
Hypoglycaemic events	5	1	1
**1st whole day after the exercise test**			
Mean glucose concentration	8.4 ± 0.4	8.8 ± 0.5	9.6 ± 0.5*
Time below range	18 [0-55]	0 [0-26]*	3 [0-35]
Time in range	1053 ± 64	1023 ± 70	853 ± 73*†
Time above range	355 ± 69	387 ± 71	552 ± 81*†
Hypoglycaemic events	11	8	10
**2nd whole day after the exercise test**			
Mean glucose concentration	9.7 ± 0.5	9.4 ± 0.5	8.5 ± 0.5*
Time below range	5 [0-44]	0 [0-41]	28 [4-65]*†
Time in range	912 ± 80	943 ± 97	998 ± 71
Time above range	555 ± 85	504 ± 99	396 ± 77
Hypoglycaemic events	13	10	20†
**Total 6 days after the exercise test**			
Mean glucose concentration	9.4 ± 0.4	9.2 ± 0.4	9.1 ± 0.4
Time below range	38 [15-164]	45 [19-231]	98 [40-366]
Time in range	5337 ± 372	5429 ± 435	5334 ± 320
Time above range	3110 ± 406	2730 ± 417	2938 ± 355
Hypoglycaemic events	60	57	92

Mean glucose concentration in mmol/l, times ranges in minutes, hypoglycaemic events as total count. CON, no dose adjustment; D40, 40% dose reduction; D20-P, postponement and 20% dose reduction. *$p<0.05$ vs CON; †$p<0.05$ vs D40. Data are presented as mean±SEM, median[IQR] or total count (n).

ESM Fig. 1

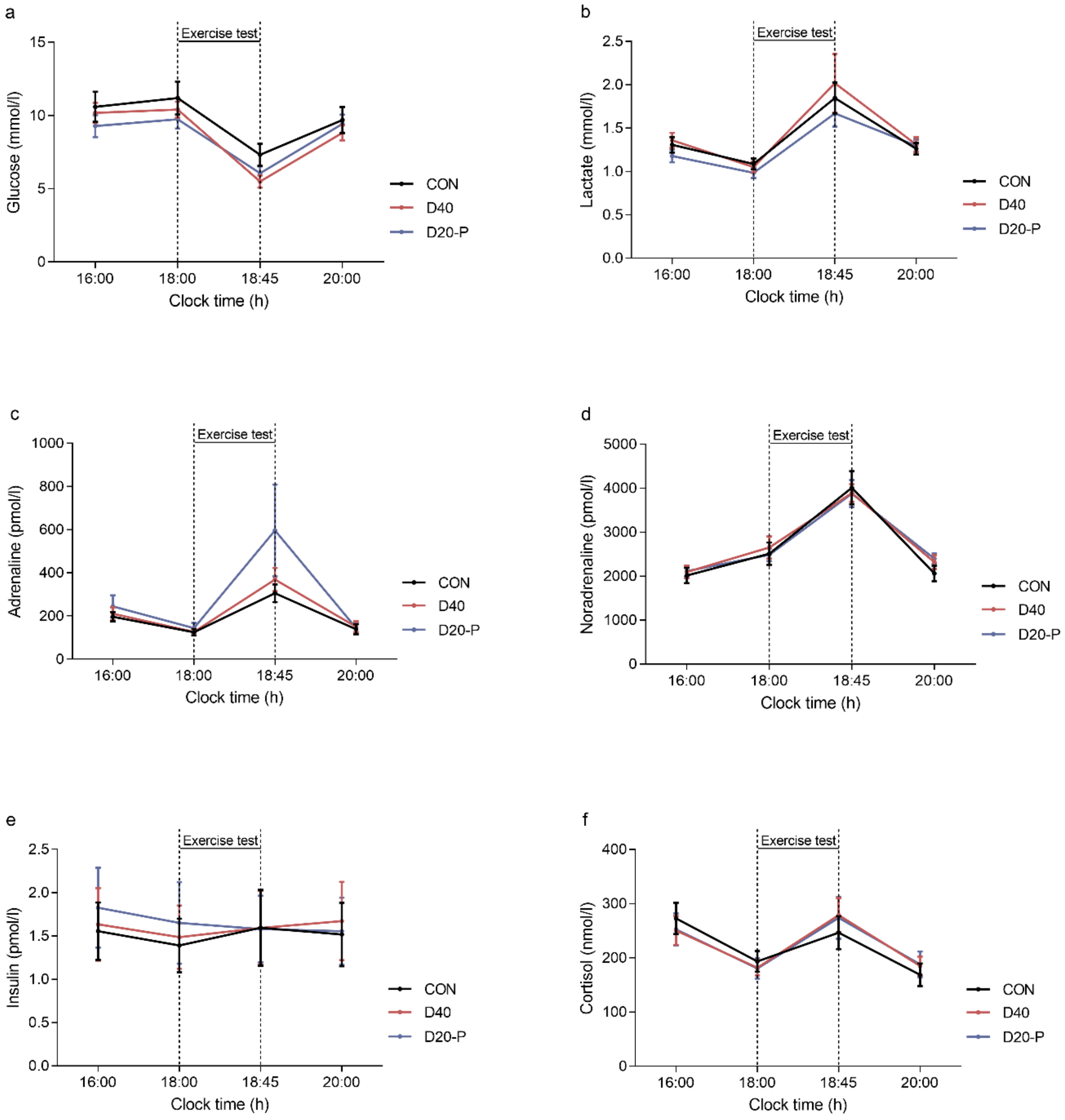

ESM Fig. 1. Concentration of glucose (a), lactate (b), adrenaline (c), noradrenaline (d), insulin (e) and cortisol (f) upon arrival (at 16:00h), before the exercise test (at 18:00h), after the exercise test (at 18.45h) and before discharge (at 20.00h), according to insulin degludec dosing regimen. CON, no dose adjustment (black); D40, 40% dose reduction (red); D20-P, postponement and 20% dose reduction (blue). Values are given as mean±SEM.

ESM Fig. 2

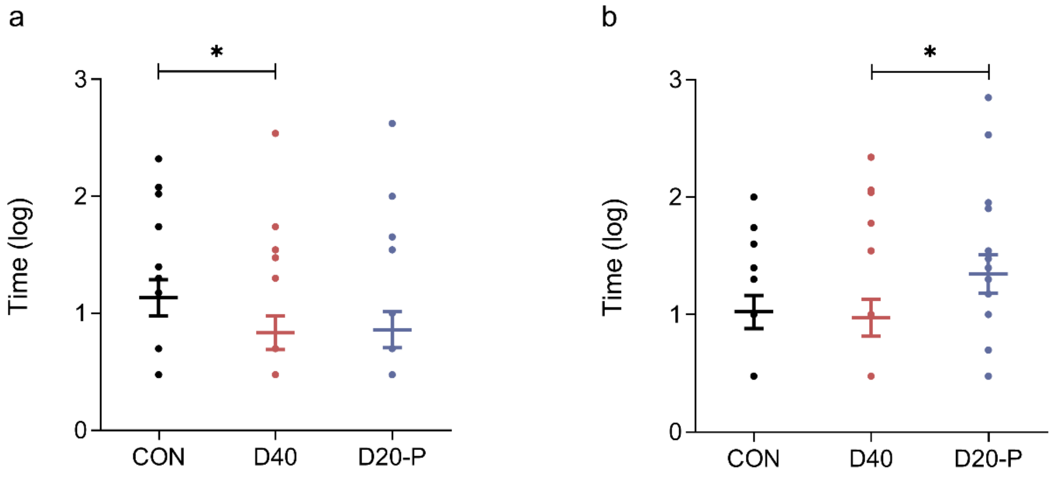

ESM Fig. 2. Time spent below range in minutes during the first (a) and second day (b) after the exercise test, according to insulin degludec dosing regimen. CON, no dose adjustment (black); D40, 40% dose reduction (red); D20-P, postponement and 20% dose reduction (blue). Values are given as individual numbers after log transformation. Bars represent mean±SEM. *Indicates $p<0.05$.

ESM Fig. 3

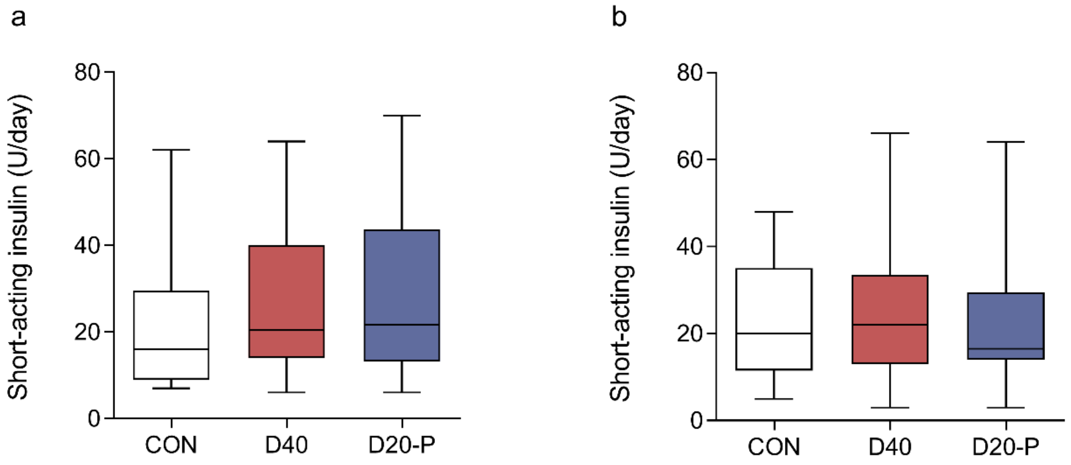

ESM Fig. 3. Total amount of short-acting insulin used on the first (a) and second day after the exercise test (b), according to insulin degludec dosing regimen. CON, no dose adjustment (white); D40, 40% dose reduction (red); D20-P, postponement and 20% dose reduction (blue). Values are given as median and interquartile range with minimum and maximum.
